# Ontario healthcare workers who sought treatment for their mental health during the first five waves of the COVID-19 pandemic: a snapshot of self-referrals across the province

**DOI:** 10.24095/hpcdp.45.2.04

**Published:** 2025-02

**Authors:** Judith M. Laposa, Duncan Cameron, Kim Corace, Heather L. Bullock, Lauren Flavelle, Natalie Quick, Karen Rowa, Sara de la Salle, Katherin Creighton-Taylor, Alice Strachan, Stephanie Carter, Paul Kurdyak, Vanessa Saldanha, Randi E. McCabe

**Affiliations:** 1 Centre for Addiction and Mental Health, Campbell Family Mental Health Research Institute, Toronto, Ontario, Canada; 2 Department of Psychiatry, University of Toronto, Toronto, Ontario, Canada; 3 St. Joseph’s Healthcare Hamilton, Hamilton, Ontario, Canada; 4 Royal Ottawa Mental Health Centre, Ottawa, Ontario, Canada; 5 Department of Psychiatry, University of Ottawa, Ottawa, Ontario, Canada; 6 Waypoint Centre for Mental Health Care, Penetanguishene, Ontario, Canada; 7 Department of Health Research Methods, Evidence and Impact, McMaster University, Hamilton, Ontario, Canada; 8 Ontario Shores Centre for Mental Health Sciences, Whitby, Ontario, Canada; 9 Department of Psychiatry and Behavioural Neurosciences, McMaster University, Hamilton, Ontario, Canada; 10 Ontario Health, Toronto, Ontario, Canada; 11 Institute for Clinical Evaluative Sciences, Toronto, Ontario, Canada

**Keywords:** healthcare worker, COVID-19, mental health, treatment, anxiety, depression

## Abstract

**Introduction:**

Healthcare workers (HCWs) have reported COVID-19 pandemic-related adverse mental health impacts. We examined the demographic profile of HCWs who self-referred for mental health treatment, how referrals changed over time in relation to waves of COVID-19, what the main problem was for which HCWs sought treatment, and how this changed during the pandemic.

**Methods:**

Five major healthcare institutions provided mental health supports to HCWs across Ontario during the pandemic. Data from May 2020 to March 2022 were collected from 2725 HCW self-referrals regarding referral frequency, main presenting mental health problem and demographic information including ethnicity, gender, age, healthcare setting, profession and whether the HCW had a prior mental health diagnosis or had received prior mental health treatment.

**Results:**

Treatment-seeking HCWs who self-referred predominantly self-identified as female and White. Almost half were nurses, and almost half had received previous mental health treatment; a slightly higher percentage reported a prior mental health diagnosis. Over 60% of the overall sample of HCWs worked in hospitals. The timing of increases and decreases in monthly new referrals roughly aligned with the onset and ending, respectively, of COVID-19 waves. The top five most common presenting problems for treatment-seeking were generalized anxiety/worry symptoms, depression, situational crisis/acute stress response, difficulty with stress/occupational or financial, and posttraumatic stress symptoms.

**Conclusion:**

Ontario HCWs self-referred to access mental health supports during the COVID-19 pandemic. The majority sought treatment for generalized anxiety/worry or depression symptoms. Results of this study may inform system planning for future pandemics, as well as for HCW wellness programs for continued workplace stress in the postpandemic period.

**Highlights**

- Ontario HCWs accessed flexibly available, low-barrier mental health supports during the COVID-19 pandemic.
- Ontario HCWs most commonly accessed mental health supports during the COVID-19 pandemic for generalized anxiety/worry symptoms and depression symptoms.
- The timing of increases and decreases in monthly new referrals roughly aligned with the onset and ending, respectively, of COVID-19 waves.
- Treatment-seeking HCWs who selfreferred predominantly were nurses, worked in a hospital setting and self-identified as female; almost 40% of participants identified as belonging to a racialized group.

## Introduction

In addition to the physical toll of the COVID-19 pandemic, there have been widespread negative mental health effects on Canadians.^1^ These effects were even more pronounced in healthcare workers (HCWs). Eighty-four percent of HCWs in the United Kingdom reported higher psychological distress than the general public during the pandemic,^2^ and a systematic review of research from around the world reported depression in 33% and anxiety in 42% of HCWs.^3^

HCWs faced unique stressors. Canadian HCWs have reported eight themes of stressful events, including managing patients dying alone; administering care perceived to be futile; feeling their professional opinions were disregarded; observing patient harm; experiencing bullying, violence and divided professional opinions; issues with resources and personal protective equipment; increased workload in the context of staffing shortages; and being in situations where personal and institutional values were conflicting.^4^ These themes were also consistent across HCWs at the global level.^5^ Many HCWs reported discrimination and/or stigma due to working with patients infected with COVID-19^6^ and this was compounded by the impact of social distancing restrictions. Redeployment in healthcare settings also led some to feel inadequately prepared for new work assignments. 

Mental health symptoms have been well documented among HCWs during the COVID-19 pandemic, and include anxiety, distress, stress, insomnia, depression, health anxiety/somatization, posttraumatic stress symptoms and fears about COVID-19.^7^-^10^ In addition, many correlates of mental health symptoms were directly related to unique aspects of HCWs’ work, including higher COVID-19 infection risk,^11^ fears of exposing family to COVID-19,^7^ increased workload and separation from family members.^12^

Individual and demographic characteristics were also associated with HCWs’ mental health during the pandemic. Nurses were more negatively affected than other professionals and had the highest level of burnout.^12^ Females tend to be overrepresented in studies of HCW distress during the pandemic^7^ and in treatment studies,^8^,^13^ although this may not be surprising given that nurses are still predominantly female (91% of regulated nurses in Canada were female in 2021^14^) and nurses represent the largest proportion of HCWs in most studies.^8^ Younger age predicted worse mental health symptoms in HCWs during the pandemic,^15^ as did history of mental health disorder,^8^ being frontline staff and female gender.^16^ These findings underscore the variability in adverse mental health impacts across a range of individual variables. 

Mental health challenges also varied during the pandemic, as each wave of COVID-19 was associated with different public health measures, individual contextual factors and impacts on the healthcare workplace. During July to December 2021, for example, symptoms of fatigue, stress/burnout, insomnia, absenteeism, functional impairment and quality of life worsened in HCWs.^17^ Depression and health anxiety/somatization were more frequent in HCWs at the COVID-19 peak compared to the initial phase,^10^ and depression and anxiety symptoms reported by HCWs lessened as the epidemic eased.^18^ Researchers examining HCWs four times over 12months found emotional exhaustion and psychological distress peaked in spring 2021, and neither rose monotonically.^9^ Emotional exhaustion decreased during periods of low rates of COVID-19-related hospitalizations and new community cases.^9^ HCW self-referral for psychiatric care was highest at the start of the pandemic, whereas psychological care requests increased in the summer of 2020.^19^ One study reported that treatment referral waves corresponded to COVID-19 community case waves, in which the highest levels of referrals were in May 2020, January 2021 and May 2021.^19^

More research is necessary to understand how mental health challenges reported by HCWs, and the extent of their treatment seeking, fluctuated over the phases of the COVID-19 pandemic. The majority of research outlined above occurred outside of North America, few studies involved Canadian HCWs and many longitudinal studies were restricted to durations of 6 to 18 months. Further, much of the research did not involve treatment-seeking HCWs, who may differ from those solely responding to general surveys about their mental health. Research in the above areas may inform system planning for future pandemics, and for worker wellness planning in healthcare organizations during continued workplace stress in the postpandemic period (e.g. critical staffing shortages, supply chain challenges of medications and equipment, etc.) 

Our study examined the following in Ontario HCWs over a 22-month period: 

(1) Demographics. What is the demographic profile of HCWs who self-referred for treatment, including race/ethnicity, gender, age, health care setting, profession and prior mental health diagnosis and/or treatment?

(2) Trends in help seeking behaviour. How many HCWs self-referred for mental health support, and did the degree of help-seeking change over time in relation to the COVID-19 waves? Did health care setting and profession vary over time?

(3) Clinical presentations. For which main presenting problems did HCWs seek treatment, and did this change over time?

## Methods

***Ethics approval***

This research was approved by the hospitals’ Research Ethics Boards (CAMH REB #086/2020, consent form requirement waived; SJHH REB #12842, written consent obtained) or exemption was provided (Ontario Shores, Royal, Waypoint).

***Mental health supports for HCWs—recruitment***

Five major healthcare institutions were funded by the Ontario government to provide mental health services to frontline HCWs in healthcare and community care settings across the province. The five hospitals offered self-referral, rapid access to free, confidential, mostly virtual services provided by trained multidisciplinary mental health professionals. Services were developed to support coping with COVID-19-related stress and its impacts on personal well-being, and were available to individuals identifying as working in a healthcare or community care setting. The five hospitals agreed on common approaches to intake and assessment, and hospital representatives regularly met as a working group to address program needs and ways to meet them.

This initiative was complementary to other local, regional and national programs and services. The initiative was advertised broadly both within the institutions and externally (via social media, hospital websites and regional outreach efforts with partners and stakeholders). HCWs could access the program by connecting to one of the five hospitals through their website or by phone. The treatment was offered virtually and in person, in English, French or other languages, using interpretation services as needed. 

***Participants***

Data were collected from 2725 HCW self-referrals across Ontario, Canada, who self-referred for treatment for their mental health during the pandemic. The five hospitals involved in the HCW initiative were: The Centre for Addiction and Mental Health (CAMH, n=1124), St. Joseph’s Healthcare Hamilton (SJHH, n=595), The Royal Ottawa Mental Health Centre (Royal, n=585), Waypoint Centre for Mental Health Care (WCMH, n=261) and Ontario Shores Centre for Mental Health Sciences (Ontario Shores, n=160). Demographic characteristics for the treatment-seeking HCWs can be seen in Table 1 by hospital site, and overall findings are listed in the Results section. 

**Table 1:** Demographics of HCW self-referrals for mental health treatment during the COVID-19 pandemic at five health care institutions, May 2020 to March 2022, Ontario, Canada

Variable	CAMH	Royal	Ontario Shores	SJHH	WCMH
*N*	Frequency (%)	*N*	Frequency (%)	*N*	Frequency (%)	*N*	Frequency (%)	*N*	Frequency (%)
Total referrals	1124	—	585	—	160	—	595	—	261	—
Age, mean (SD)^a ^	1087	34.2 (10.7)	539	35.4 (12.6)	158	37.2 (11.4)	514	36.7 (10.7)	243	39.7 (11.4)
Missing	37	—	0	—	2	—	81	—	18	—
Self-reported gender	1124	—	431	—	116	—	520	—	242	—
Female	978	87.0	365	84.7	107	92.2	455	87.5	211	87.2
Male	136	12.1	57	13.2	9	7.8	62	11.9	29	12.0
Genderqueer/non-binary	1	0.8	1	0.2	NA	NA	3	0.6	2	0.8
Prefer not to disclose	9	0.1	8	1.9	NA	NA	NA	NA	NA	NA
Missing	0	—	108	—	44	—	75	—	19	—
Ethnicity	366	—	427	—	116	—	511	—	176	—
Asian (East, South, Southeast)	95	26.0	16	3.7	21	18.1	34	6.7	5	2.8
Black	14	3.8	14	3.2	2	1.8	8	1.6	4	2.3
Indigenous	NA	NA	7	1.6	1	0.9	NA	NA	3	1.7
Latin American	NA	NA	2	0.4	1	0.9	8	1.6	3	1.7
Middle Eastern	16	4.4	4	0.9	5	4.3	7	1.3	1	0.6
Mixed heritage	20	5.6	11	2.6	5	4.3	7	1.3	4	2.3
White	200	54.6	341	79.9	80	69.0	216	42.3	146	83.0
Other	21	5.7	32	7.5	1	0.9	231	45.2	10	5.7
Missing	758	—	112	—	44	—	84	—	85	—
Past mental health diagnosis	1123	—	432	—	NA	NA	375	—	138	—
Yes	530	47.2	216	50.0	NA	NA	198	52.8	95	68.8
No	593	52.8	216	50.0	NA	NA	177	47.2	43	31.2
Missing	1	—	107	—	NA	NA	220	—	123	—
Received past treatment	1123	—	431	—	NA	NA	361	—	132	—
Yes	579	51.6	292	67.7	NA	NA	143	39.6	96	72.7
No	544	48.4	139	32.3	NA	NA	218	60.4	36	23.3
Missing	1	—	108	—	NA	NA	234	—	129	—

**Abbreviations: **CAMH, The Centre for Addiction and Mental Health; HCW, healthcare worker; NA, not available; Ontario Shores, Ontario Shores Centre for Mental Health Sciences; Royal, The Royal Ottawa Mental Health Centre; SD, standard deviation; SJHH, St. Joseph’s Healthcare Hamilton; WCMH, Waypoint Centre for Mental Health Care. 

**Notes:** Data are reported as available with *n* reported for each variable. Previous treatment and previous diagnosis data were unavailable for Ontario Shores Mental Health Services. 

^a^ Age is approximated based on year of birth only. 

Each hospital site provided a variety of mental health services for HCWs, including internet-based cognitive behavioural therapy, single-session psychotherapy, a brief course of coping-focussed psychotherapy (4–8 sessions depending on site), medication consultation and various other services. The data for this paper include HCWs self-referring for any type of treatment. 

***Measures***

**Referral frequency**

Each site calculated the total number of cumulative referrals, as well as monthly new referrals over time. 

**Demographic information**

Participants reported race/ethnicity, gender, age, healthcare setting, profession, whether they had a prior mental health diagnosis (yes/no) and whether they had received prior mental health treatment (yes/no). The assessing clinician reported one main presenting problem (selected from a list); only the Royal site included all problems that applied. 

***Procedure***

Data were collected separately by each institution and sent monthly to a coordinator who maintained a master database for storage and analysis. The five institutions launched their HCW initiatives on different dates between early April (CAMH, WCMH, Ontario Shores) and May (SJHH, Royal) 2020. The present analysis describes data collected from May 2020 to the end of March 2022, by the beginning of the sixth COVID wave in Ontario. In Ontario, wave 1 took place from mid-March to mid-July 2020; wave 2 from mid-October 2020 to mid-February 2021; wave 3 from early April to mid-July 2021; wave 4 from mid-August to late October 2021; and wave 5 from mid-December 2021 to late February 2022).^20^

**Data analysis**

Data were collected cumulatively and without unique identifiers, precluding analysis either between or within individuals. Therefore, all analyses presented are descriptive in nature. 

## Results

Core demographic variables for each of the five sites are presented in Table 1.

The overall sample was predominantly female (87.0%) and White (61.6%), with a mean age of 36.33 (SD=10.49). Approximately half (50.2%) of the individuals who self-referred had received previous treatment for a mental health issue, and a slightly higher percentage (54.2%) had received a past formal diagnosis of a mental health condition. Table 1 shows the self-referrals breakdown by hospital site, over the period of May 2020 to March 2022.

***Total referrals***

**Figure 1:**
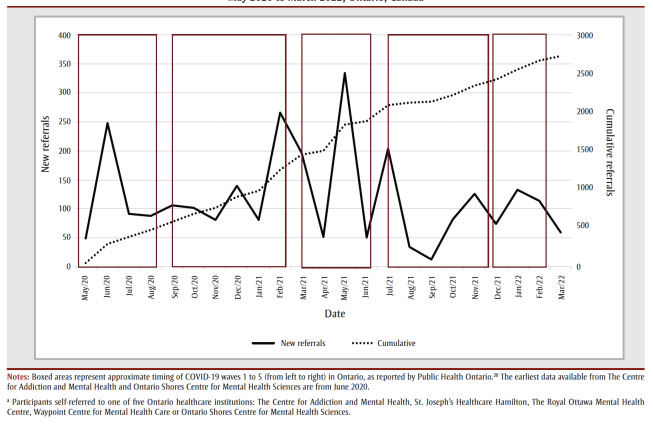
Cumulative and monthly new self-referrals of healthcare workers seeking mental health treatment during the COVID-19 pandemic, May 2020 to March 2022, Ontario, Canada

Figure 1 represents the cumulative referrals to all five sites from May 2020 to March 2022, and the change in new referrals seen each month as the COVID-19 pandemic progressed from the end of the peak of the first wave to the beginning of the sixth wave in Ontario. The mean monthly referrals over the entire period was 118.5 (SD=80.64), with a range of 13 (in September 2021) to 334 (in May 2021). As represented in Figure 1, the timing of increases and decreases in monthly new referrals roughly aligned with the onset and ending, respectively, of COVID-19 waves, indicating greater seeking of mental health services by health professionals as COVID-19 cases increased.

***Presenting problem***

**Figure 2:**
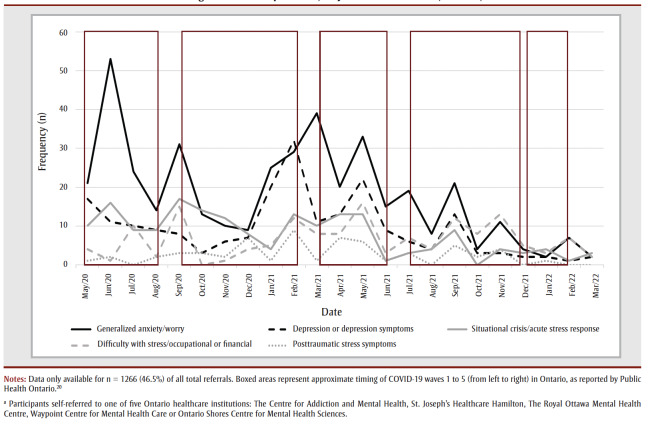
Monthly new self-referrals for the five most commonly reported presenting problems of healthcare workers seeking mental health treatment during the COVID-19 pandemic, May 2020 to March 2022, Ontario, Canada

Data on presenting problem were available for n=1266 of the total referrals. Figure 2 displays frequencies for monthly new referrals of the top five most common presenting problems seen upon initial referral across all sites. As seen in Figure 2 generalized anxiety/worry symptoms were the most prevalent, with 414individuals, who accounted for 32.7% of the total referrals. Depression was the next most common, with 214 individuals, who accounted for 16.9% of the total cumulative referrals. The next most common presenting problems were situational crisis/acute stress response (n=180; 14.2%), difficulty with stress/occupational or financial (n=150; 11.9%) and posttraumatic stress symptoms (n=60; 4.7%). These were consistently the top five presenting problems over the entire period. 

Although they were reported, symptoms of health anxiety (1.2% of the sample) and difficulty with stress/positive testing for COVID-19 (2.0% of the sample) were relatively infrequent. Other low frequency presenting problems included obsessive-compulsive symptoms (reported by 0.9% of the sample), adjustment disorders (reported by 2.2% of the sample), substance use disorder symptoms (reported by 1.8% of the sample), alcohol use disorder symptoms (reported by 1.0% of the sample), avoidance due to anxiety symptoms (reported by 0.5% of the sample), symptoms of bereavement/grief and loss (reported by 0.6% of the sample) and symptoms of social anxiety disorder (reported by 1.0% of the sample). Finally, a further n = 105 individuals or 8.3% of the sample reported miscellaneous/other symptoms (e.g. insomnia, eating disorder symptoms, difficulty with relationships/family).

***Profession***

**Figure 3:**
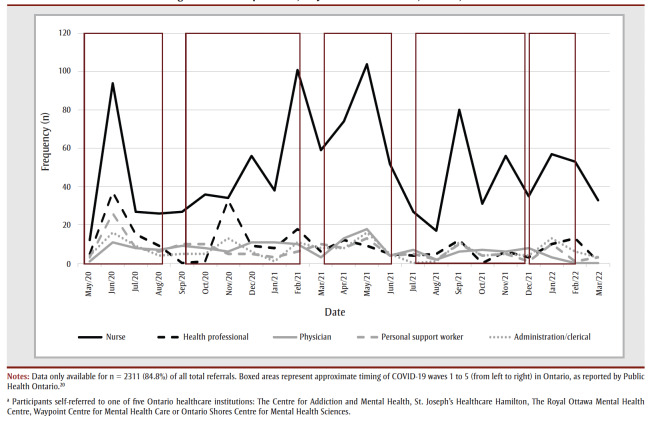
Monthly new self-referrals for the five most commonly reported professions of healthcare workers seeking mental health treatment during the COVID-19 pandemic, May 2020 to March 2022, Ontario, Canada

Data on profession were available for n=2311 of the total referrals. Figure 3 represents the monthly new self-referrals for each of the five most common professions observed among all referrals. As seen in Figure 3, nurses were highly represented in the sample relative to other professions, making up n=1129 (48.9%) of the total cumulative referrals; the next most common professions were health professionals (n=221; 9.6%), physicians (n=159; 6.9%), personal support workers (n=158; 6.8%) and administrative/clerical staff (n=158; 6.8%). Social workers were also relatively well represented in the sample, at n = 103 (4.5%) of the total cumulative referrals. Community service/support workers (n=96; 4.2%) and facilities/environmental service employees (n=48; 2.1%) were also seen with less frequency. A further n=239 individuals (10.3%; e.g. chaplains, laboratory technicians, research personnel, or other professions) were referred from other areas of healthcare institutions.

***Setting***

**Figure 4:**
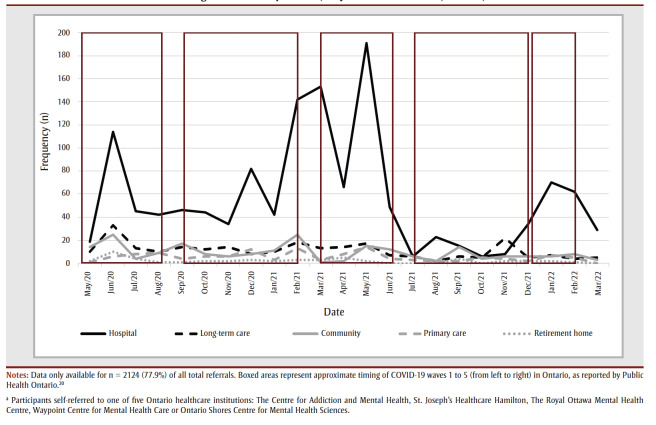
Monthly new self-referrals for the five most commonly reported healthcare settings, whose healthcare workers sought mental health treatment during the COVID-19 pandemic, May 2020 to March 2022, Ontario, Canada

Data on participants’ healthcare setting were available for n=2124 individuals. Figure 4 represents the monthly new referrals for the five most commonly reported healthcare settings in which individuals were working at the time of referral. As seen in Figure 4, referrals came most frequently from hospitals by a large margin, with n=1322 individuals, representing 62.2% of the total cumulative referrals. Of these, 570 (43.1%) were working in inpatient wards, with a further n=291 (22.0%) working in intensive care units. The majority of the remaining HCWs who self-referred were working in long-term care (n=255; 12.0%), community care centres (n=212; 10.0%), primary care (n=127; 6.0%) or retirement homes (n=51; 2.4%). The less frequently observed healthcare settings included home and community care (n=13; 0.6%) and public health (n= 4; 0.2%), with the remaining n=140 (6.6%) working in other healthcare or related settings.

## Discussion

We examined the demographic profile of treatment-seeking HCW self-referrals during the COVID-19 pandemic, the mental health problem for which they sought treatment and how treatment referrals fluctuated over 22 months of the COVID-19 pandemic. The most common presenting concern was generalized anxiety/worry symptoms. The timing of increases and decreases in monthly new referrals roughly aligned with the onset and ending, respectively, of COVID-19 waves. 

The demographic data revealed that the HCWs who self-referred for treatment were predominantly female, White and nurses, and this was not unique to the Canadian context. These findings are consistent with a larger body of research showing that nurses were more negatively impacted during the pandemic than other professions^12^ and had high burnout levels;^9^ and that nurses, and females more generally, tend to seek treatment more often.^8^,^13^ However, it is also true that the majority of healthcare staff in hospital settings are nurses, and that nurses are predominantly female.^14^ Physicians were a small but significant referral stream. 

The average age of treatment-seeking HCWs was in the mid-thirties, in line with multiple studies showing an association between younger age and greater mental health symptoms among HCWs during the pandemic.^7^ Approximately half of the HCW self-referrals had received previous treatment for a mental health issue, and reported a prior mental health diagnosis. This finding is similarly consistent with previous work,^8^,^21^,^22^ and also highlights the large number of HCWs who sought treatment for the first time during the pandemic. Almost two-thirds of treatment-seeking HCW self-referrals came from hospital settings, in keeping with the high emotional toll of being a frontline HCW.^16^ Approximately 10% of HCW self-referrals in hospital support positions (administration/clerical, facilities/environmental) sought treatment, highlighting the importance of mental health treatment programs for HCWs being inclusive of a variety of professional roles. Overall, these findings underscore the variability in the adverse mental health impacts of the pandemic across a range of individual variables, and are markedly consistent with findings of prior research. 

Our study identified types of mental health issues for which HCWs self-referred for treatment. In concordance with systematic reviews showing the ubiquitous nature of anxiety and depression symptoms among HCWs during the pandemic,^3^ the top two most common presenting problems for which HCWs sought treatment were generalized anxiety/worry symptoms and depression, with these two areas representing half of the HCWs self-referrals in this study. This Canadian finding is in contrast to research done in other countries, which found that depression (Mexico^10^) or distress (US^7^), instead of anxiety, was the most common reason for seeking treatment. A meta-review of systematic reviews found that during the pandemic in the United Kingdom, HCWs most commonly reported anxiety as the reason for seeking treatment, whereas in the Eastern Mediterranean region the presenting problem was most often stress, and in the Middle East, HCWs most often reported depression as the reason for seeking treatment.^23^

Notably, in our study, treatment seeking for worry symptoms peaked in the first COVID-19 wave, whereas treatment seeking for depression symptoms did not peak until late in the second wave. Throughout the 22 months, very rarely did the demand for depression-related treatment surpass the demand for treatment for generalized anxiety/worry. During the pandemic, there was a marked increase in research on HCWs’ mental health. Pre-pandemic, the majority of the research in this area involved physicians, nurses and emergency services workers. In a pre-pandemic study of over 37000 HCWs (representing several occupations) in the US, insufficient sleep (41%) and depression (18.9%) were the most common conditions, although the study did not assess anxiety.^24^ It is nevertheless interesting to contrast this to our study’s finding that during the pandemic 32.7% sought treatment for generalized anxiety/worry and 16.9% for depression. 

The three next most common presenting problems were situational crisis/acute stress response, difficulty with stress/occupational or financial and posttraumatic stress symptoms. These findings supplement cross-sectional studies of symptoms reported by HCWs, with information on problems for which HCWs actually sought treatment. This is vital, as many affected HCWs do not seek treatment. Future studies could include burnout and moral injury as specific presenting concerns.

With respect to changes in HCW self-referrals over time, on average, over 100 HCWs self-referred each month over the course of the pandemic. However, there was a large range, with a high of 334 in May 2021, down to a low of 13 in September 2021. The timing of increases and decreases in monthly new referrals roughly aligned with the onset and ending, respectively, of COVID-19 waves, indicating greater seeking of mental health services as COVID-19 cases increased. 

There is convergence with prior Canadian HCW studies, namely that psychological distress and emotional exhaustion peaked in spring 2021, and the latter decreased during periods of low COVID-19 hospitalization and community case rates,^9^ and that two of the three highest HCW treatment referral rates were in May 2020 and May 2021.^19^ In contrast to Sheehan et al.,^19^ who found a high referral peak in January 2021, we found the second highest referral rate occurred in March 2021, slightly after the end of the second wave of the pandemic. 

Despite the ongoing nature of the pandemic, it was striking to see a large drop in HCW self-referrals in the fourth and fifth waves of the pandemic, when there were lower referral rates than in the first three waves. This may be partially explained by the more widely available COVID-19 vaccinations, the increased knowledge of COVID-19 and how to manage it, and the easing of some government and hospital physical distancing restrictions by that time. 

There were several system-level lessons learned from this multihospital initiative, which was mobilized within weeks of the pandemic onset. First, as the HCW program was designed to cover the province and had a standardized intake process, the totality of resources across the five sites could be shared or distributed across the province. Second, providing virtual services further facilitated the sharing of resources, and had two significant benefits. One benefit was that HCWs had access to services outside their home institution, thus facilitating a greater degree of confidentiality. HCWs often preferred to access programming outside of their own local area (even though it was virtual), and staff of the five hospitals often sought care from facilities other than where they worked. The other benefit was that having the hospitals work together and share intake responsibilities enabled timely access to care even when the demand rose; wait times were kept as low as possible by pooling resources and monitoring waits, ensuring rapid treatment access (from no to very short wait-times). The virtual format meant no matter where people lived, if they had phone or internet access, they could access services equitably. Third, self-referral removed an access barrier. 

Despite other support programs being available, such as Employee Assistance Programs, the hospital initiatives were well accessed. The sector-specific nature (i.e. the healthcare sector) of the care that was provided was an important asset, and should be present in mental health support programs for HCWs. The clinicians’ ability to understand the context of the work and the types of challenges that HCWs were experiencing was a key to success. Anecdotally, it is likely that the services of this program enabled HCWs to continue to work as opposed to taking a leave, and those who did take a leave still required support as they returned to the workplace. As with other healthcare services, human resources was a challenge, with programs sometimes needing to “borrow” clinicians from other areas to meet the need posed by the HCW treatment program. 

***Strengths and limitations***

Strengths of the current study include its multisite nature, the large sample of treatment-seeking HCWs, the collection of data throughout a 22-month period and the ability to compare treatment-seeking referrals to community COVID-19 waves. 

Limitations include that the data were collected without unique identifiers, which precluded analysis between or within individuals. The study therefore combined all treatment-seeking HCWs; unique identifiers would have been needed to explore which HCWs requested the different types of mental health assistance, why they chose the type of treatment they did and the outcomes of those various treatment options (see Laposa et al.,^8^ for an evaluation of the HCW brief treatment). 

Although the sample in this study is large, some data are missing, and many HCWs whose mental health was negatively affected during the pandemic may not have self-referred for treatment, to this program or to something else. Theoretically, a HCW could have self-referred for treatment more than once; however, anecdotal reports from staff would indicate that it was very rare, as once the HCWs were enrolled in treatment, if they needed something more than the brief intervention, they would be connected to one of the clinic program standard pathways, or to other community resources. Finally, the majority of the hospital sites reported that only 12% of the treatment-seeking HCW self-referrals were male; therefore, the findings may not be representative of this group. 

More work is needed to understand which HCWs do not seek treatment and why, so that programs can be tailored with successful outreach initiatives.

## Conclusion

This study provides a profile of HCWs who self-referred for mental health supports during the pandemic through a coordinated, rapid-access service provided by five major hospitals in Ontario. The majority of HCWs who accessed the service were female (as are most HCWs), came from a nursing background and had prior mental health diagnoses and/or prior treatment. When setting up similar services in the future, based on treatment-seeking patterns, one might particularly consider a target audience of these groups. 

The structure of the program allowed for more equitable and timely access to services by virtually “pooling” the resources across the five sites. The virtual delivery of care meant that access and wait times were not dependent upon where one lived in the province, and conferred the further benefit of prioritizing participant choice of treatment site, which was particularly important when that individual worked at one of the sites providing mental health treatment and preferred to go elsewhere to preserve privacy or confidentiality. 

Finally, identifying the top presenting concerns of the HCWs who self-referred in this study—namely, generalized anxiety/worry, depression, situational crisis/acute stress response, difficulty with stress/occupational or financial, and posttraumatic stress symptoms—may inform planning for ongoing HCW mental health supports in the post-pandemic period. The study findings suggest we should expect HCWs to seek treatment for their mental health during outbreak conditions, and highlight the need for corresponding supports to be available for HCWs during those times.

## Acknowledgements

We would like to thank the HCW participants for their fantastic service during the pandemic. Thanks also go to the hospital administrations for rapidly developing HCW supports soon after the COVID-19 onset, as well as to the government of Ontario, for funding these crucial services for HCWs. 

***Funding***

This research received no specific grant from any funding agency in the public, commercial or not-for-profit sectors.

## Conflicts of interest

The authors declare that there are no conflicts of interest.

## Authors’ contributions and statement

JL, RM, DC: conceptualization.

JL, RM, DC, KC, KR, SDLS, LF, KCT, NQ, VS, SC: implementation.

JL, RM, DC: analysis.

JL, RM, DC: writing—original draft.

JL, RM, DC, KC, KR, SDLS, LF, KCT, NQ, VS, SC, PK, HB, AS: writing—review and editing.

The content and views expressed in this article are those of the authors and do not necessarily reflect those of the Government of Canada.
